# Matched Sizes of Activating and Inhibitory Receptor/Ligand Pairs Are Required for Optimal Signal Integration by Human Natural Killer Cells

**DOI:** 10.1371/journal.pone.0015374

**Published:** 2010-11-05

**Authors:** Karsten Köhler, Shiqiu Xiong, Joanna Brzostek, Maryam Mehrabi, Philipp Eissmann, Alice Harrison, Shaun-Paul Cordoba, Stephane Oddos, Vladimir Miloserdov, Keith Gould, Nigel J. Burroughs, Philip Anton van der Merwe, Daniel M. Davis

**Affiliations:** 1 Division of Cell and Molecular Biology, Imperial College London, London, United Kingdom; 2 Sir William Dunn School of Pathology, Oxford University, Oxford, United Kingdom; 3 Wright-Fleming Institute, Imperial College London, London, United Kingdom; 4 Warwick Systems Biology Centre, University of Warwick, Coventry, United Kingdom; Centre de Recherche Public de la Santé (CRP-Santé), Luxembourg

## Abstract

It has been suggested that receptor-ligand complexes segregate or co-localise within immune synapses according to their size, and this is important for receptor signaling. Here, we set out to test the importance of receptor-ligand complex dimensions for immune surveillance of target cells by human Natural Killer (NK) cells. NK cell activation is regulated by integrating signals from activating receptors, such as NKG2D, and inhibitory receptors, such as KIR2DL1. Elongating the NKG2D ligand MICA reduced its ability to trigger NK cell activation. Conversely, elongation of KIR2DL1 ligand HLA-C reduced its ability to inhibit NK cells. Whereas normal-sized HLA-C was most effective at inhibiting activation by normal-length MICA, only elongated HLA-C could inhibit activation by elongated MICA. Moreover, HLA-C and MICA that were matched in size co-localised, whereas HLA-C and MICA that were different in size were segregated. These results demonstrate that receptor-ligand dimensions are important in NK cell recognition, and suggest that optimal integration of activating and inhibitory receptor signals requires the receptor-ligand complexes to have similar dimensions.

## Introduction

NK cell activation is regulated by a balance of signals from various germ-line encoded activating receptors and inhibitory receptors [1], [2]. One of the best studied activating receptors is the lectin-like receptor NKG2D, which recognises proteins that are usually not expressed on cells but are upregulated in response to cellular stress [3], [4], [5], [6]. Signaling downstream from activating receptors commonly involves the phosphorylation of the immunoreceptor-tyrosine based activation motifs (ITAMs). NKG2D signals via an adapter DAP10 that contains an ITAM-like motif (YXNM), which can be phosphorylated by Src-family kinases [7].

Human inhibitory NK cell receptors include members of the Killer-cell Immunoglobulin-like Receptor (KIR) and the Leukocyte Ig-like receptor (LILR) families, and the CD94:NKG2A heterodimer, all of which recognise class I MHC molecules. Ligation of KIR2DL1 by a subset of class I MHC proteins that includes HLA-Cw4 and -Cw6 inhibits NK cell-mediated lysis [8]. In this manner, loss of class I MHC protein from the surface, i.e. “missing self”, can render cells susceptible to NK lysis [9], [10], [11], [12]. KIR2DL1 signal transduction is mediated via immunoreceptor-tyrosine based inhibition motifs (ITIMs) which are phosphorylated by tyrosine kinases and then recruit SH2-domain containing phosphatases [13], [14].

Lymphocyte activation involves the engagement of multiple cell surface receptors with ligands presented on the surface of other cells in a contact area termed the immunological or immune synapse (IS) [15], [16], [17], [18], [19], [20]. Because lymphocyte cell surface molecules vary considerably in size it has been suggested that they may segregate according to size within the IS [21]. This hypothesis specifically suggests that receptor-ligand complexes with similar dimensions would be expected to co-localise, whereas those differing in size would be expected to segregate. Lymphocyte signaling is initiated by tyrosine phosphorylation, which is regulated by the balance between tyrosine kinases and phosphatases at the cell membrane. The kinetic-segregation model of T cell antigen recognition proposed that signaling is initiated by size-based segregation of the engaged T cell receptor (TCR) from receptor tyrosine phosphatases with large ectodomains such as CD45 and CD148, leading to phosphorylation of the TCR/CD3 complex [22]. A number of studies have provided support for this model [23]. Firstly, molecules at the T cell IS do segregate according to size [15], [16], [24], although active, cytoskeletally-driven transport processes are also involved [25]. Secondly, CD45 and CD148 are excluded from the site of TCR engagement and triggering [26], [27]. Thirdly, truncation of CD45 and CD148 ectodomains [28], [29] and elongation of TCR ligands [30], [31] abrogate TCR triggering.

However, whereas T cell activation is controlled primarily by a single receptor, i.e. the TCR, NK cells are regulated by the integration of signals from a number of activating and inhibitory receptors [32]. Thus, it is not immediately obvious how the dimensions of protein complexes would influence signal integration by multiple receptors expressed by NK cells. Intriguingly, both the inhibitory HLA/KIR and activating NKG2D/MICA complexes are small, spanning 10–15 nm [33], [34], [35], and have been shown to segregate within the IS from the larger [∼40 nm [21], [36]] integrin/ligand complex LFA1/ICAM-1, even in the absence of any active or cytoskeletally-driven process [17], [19], [37], [38].

Here we explore the role of ectodomain size in NK cell activation by manipulating the length of activating and/or inhibitory receptor ligands. Specifically, we analysed the effects of elongating MICA and/or HLA-Cw6 on NK cell activation or inhibition, and examine how size influences segregation within the NK cell IS. We show that ligand elongation abrogates both activating and inhibitory receptor function. We also demonstrate that size compatibility is important for the colocalization of, and signal integration between, engaged activating and inhibitory receptors, and that differences in ectodomain size are sufficient to drive segregation within the IS. This suggests a general principle for the importance of proteins size in how signals integrate from multiple receptor/ligand interactions across intercellular contacts.

## Results

### The size of MICA is important in triggering NKG2D-dependent lysis

**Figure 1:**
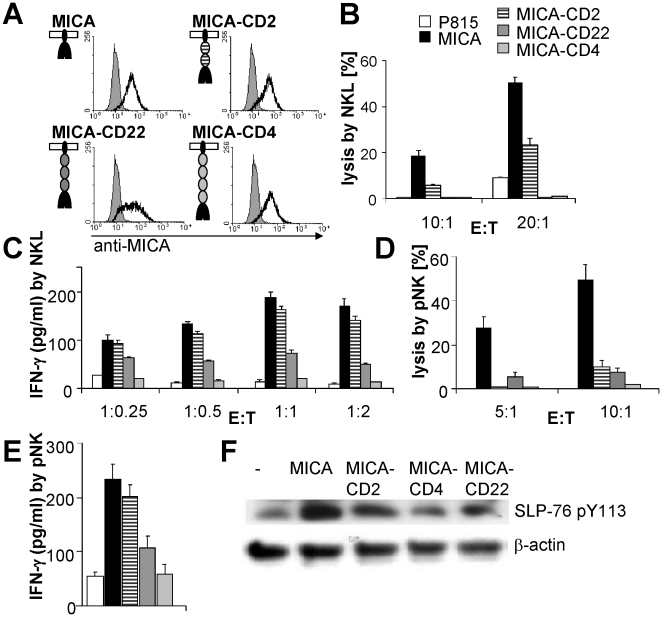
Elongation of MICA abrogates its ability to activate NK cells. (A) Normal-length and elongated forms of MICA-GFP were expressed at comparable levels in P815 cells. MICA was elongated by inserting 2, 3 or 4 Ig domains from CD2, CD22, or CD4. (B-E) Effect of MICA elongation on target cell lysis (B, D) and IFN-γ production (C, E) by NKL (B, C) or IL-2 stimulated primary NK (D, E) cells. For lysis, NK cells were incubated with [^35^S]-Met labeled P815 cells expressing normal-length or elongated MICA for 5 h at the indicated E:T ratios. Lysis percentages are expressed as [^35^S]-Met release relative to total [^35^S]-Met. The data in B and D are representative results from three experiments. For IFN-γ secretion, NK cells incubated for 16 h at the indicated E:T ratios (or E∶T = 1∶1 for panel E) with P815 cells expressing normal-length or elongated MICA. IFN-γ secretion was quantified by ELISA. Data in C and E are representative results from four and two experiments respectively. Error bars are SD of triplicate measurements. In all panels B-E the same key applies for each bar, as indicated in panel B. (F) Effect of MICA elongation on NKG2D signaling. NKL cells were stimulated by P815 cells expressing normal-length and elongated MICA for 2 min. Cell lysates were analysed by Western-blot using an antibody specific for the phospho-tyrosine 113 of SLP-76.

The size of MICA was varied by inserting 2, 3 or 4 independently-folding Ig domains from CD2, CD22, or CD4 respectively [30], [31], in the stalk region linking the transmembrane and extracellular domains (Figure 1A). These inserts were chosen because they are structurally well-characterised and have previously been shown to elongate cell surface proteins without disrupting their ligand binding properties [30], [31]. These differently-sized variants of MICA were then expressed in the mouse mastocytoma cell line P815, which does not express any ligand for human NKG2D endogenously. Transfected P815 cells were selected for comparable expression levels by fluorescence-activated cell sorting (Figure 1A) and then tested for their susceptibility to lysis by NK cells (Figure 1B). Expression of normal-length MICA was sufficient to induce lysis of P815 cells by the immortal human NK cell tumor line NKL. However, elongation of the MICA-ectodomain significantly reduced its ability to trigger lysis. Indeed, the ability of MICA to trigger NK cell cytotoxicity varied inversely with its length.

To test whether elongation affected other functional responses, we also compared the ability of normal-length and elongated MICA to trigger IFN-γ production by NKL cells. While normal-length MICA was sufficient to trigger IFN-γ, MICA elongated by 2 or 3 Ig domains was less stimulatory and MICA extended by 4 Ig domains did not elicit any IFN-γ response (Figure 1C). Similar results were obtained when we assayed intracellular IFN-γ production by flow cytometry, confirming that this effect was the result of decreased IFN-γ production rather than increased consumption (Figure S1A). Elongation of MICA also abrogated NK cell degranulation as measured by induction of CD107a expression on the cell surface (Figure S1B). Finally, elongation of MICA also inhibited target cell lysis and IFN-γ production by primary NK cells (Figure 1D, E).

In order to examine the effect of elongation of MICA on earlier activation events we also examined the phosphorylation of SLP-76, an adapter protein involved in the early stages of NKG2D signaling [39]. Incubation of NKL cells with MICA-expressing P815 cells induced SLP-76 phosphorylation within 2 min of co-incubation. The extent of phosphorylation was dramatically decreased by elongation of MICA (Figure 1F). Thus, elongation of MICA abrogates the early stages of NKG2D signaling as well as later cellular responses.

### Elongated MICA proteins bind to NKG2D

**Figure 2:**
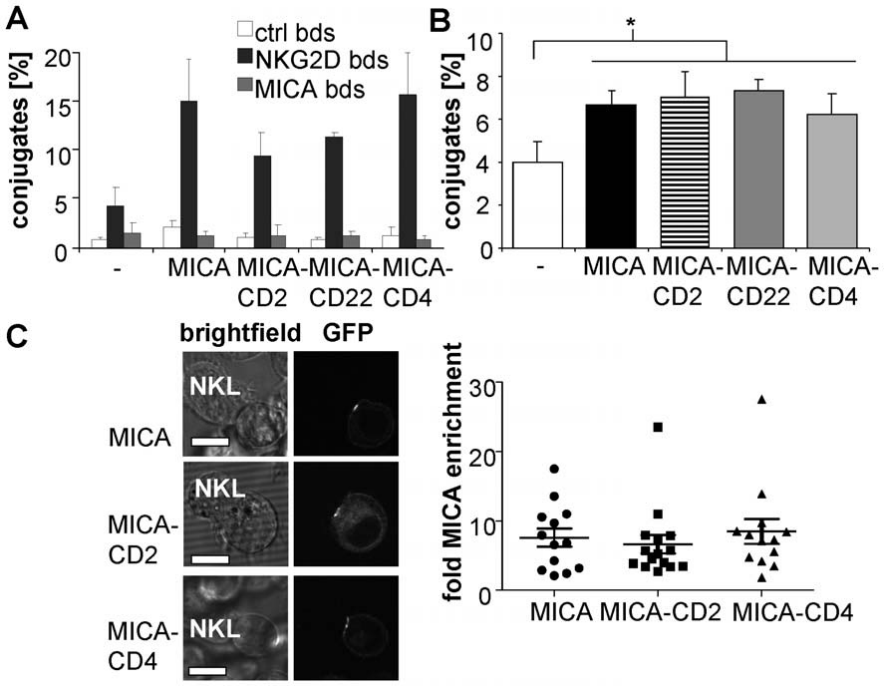
Elongated MICA binds NKG2D. (A) Binding of NKG2D beads. P815 expressing normal-length or elongated MICA were incubated with uncoated fluorescent beads, or beads coated with NKG2D-Fc or (control) MICA-Fc. Bead binding was analysed by flow cytometry and expressed as percentage of cells forming conjugates to bead. The mean (normalized to the number of conjugates for NKG2D beads to P815 MICA) and SD of three independent experiments are shown. (B) Conjugation of P815 and NKL cells. PKH67-labeled P815 cells expressing the indicated form of MICA were incubated for 30 min with the equal number of PKH26-labeled NKL cells and analysed by flow cytometry. The percentage of the NKL cells forming conjugates with P815 cells is shown. The data represents the mean and SD of three independent experiments. p = 0.04; 0.06; 0.02; 0.007 for P815 vs. MICA; MICA-CD2; MICA-CD22; MICA-CD4 respectively (one-tailed t-test). (C) Clustering of MICA in NKL immune synapses. P815 cells expressing the indicated length variant (or normal-length) of MICA-GFP were allowed to form contacts with NKG2D-expressing NKL cells for 10 min and imaged by live-cell confocal microscopy. Clustering was quantified as fold-enrichment of GFP fluorescence intensity within the contact area versus outside the contact area. Each point represents an individual cell/cell contact with the mean and SD shown. The scale bars represent 10 µm.

To test the ability of elongated MICA to be bound by NKG2D, we compared the binding of NKG2D-coated beads to P815 cells expressing normal-length versus elongated forms of MICA (Figure 2A). NKG2D-coated beads bound at similar levels to P815 cells expressing normal-length or elongated MICA both in the absence (Figure 2A) and presence (Figure S2) of a titrated blocking antibody, indicating that the different length variants of MICA had a comparable ability to bind NKG2D.

To examine whether elongated variants of MICA could bind NKG2D in the context of an intercellular contact, we used flow cytometry to measure conjugate formation between NKL cells and P815 transfectants (Figure 2B). Expression of all MICA variants on P815 cells resulted in a comparable increase in conjugate formation, indicating that the elongated forms of MICA are able to bind NKG2D in a cell-cell interface (Figure 2B).

As a final test of the ability of elongated MICA to bind NKG2D we examined their clustering in IS between NKL and P815 cells expressing variants of MICA tagged with GFP, using confocal fluorescent microscopy. Normal-length MICA-GFP accumulated at the IS, leading to a 6–8 fold increase in fluorescence intensity compared to elsewhere at the cell surface (Figure 2C). MICA elongated with 2 (MICA-CD2) or 4 (MICA-CD4) Ig domains clustered at the IS to a similar extent (Figure 2C), providing further confirmation that elongation of MICA does not abrogate binding to NKG2D at the cell-cell interface.

### Elongation of HLA-Cw6 reduces KIR2DL1-mediated inhibition

**Figure 3:**
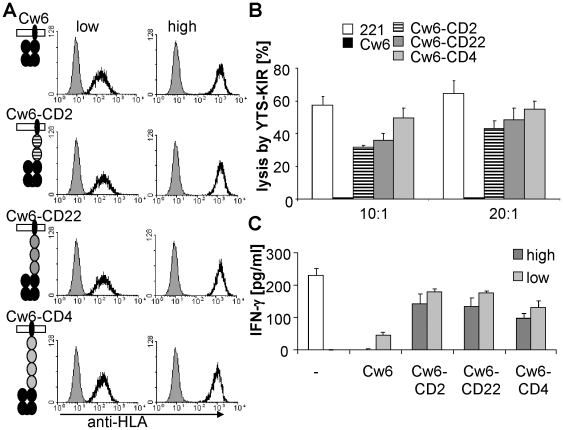
Elongation of HLA-Cw6 abrogates its inhibitory effect. (A) Normal-length and elongated forms of HLA-Cw6 were expressed on 221 cells at matched (high or low) levels. (B) 221 target cells expressing normal-length or elongated Cw6 were incubated with KIR2DL1-expressing YTS effector cells for 5 h at the indicated E:T ratios. Lysis of 221 cells was assayed as in Figure 1B. (C) YTS-KIR2DL1 cells were stimulated with 221 cells expressing no, low or high levels of the indicated form of Cw6 for 16 h. Supernatants were harvested and IFN-γ secretion quantified by ELISA. Data in B and C are representative results from four independent experiments. Error bars are SD of triplicate measurements.

Since the length of MICA was shown to be important for NKG2D-dependent activation, we next examined whether recognition by inhibitory receptors was also influenced by the size of their ligands. For this we used the well-characterised cell line YTS, which is activated principally via engagement of the 2B4 and CD28 receptors [40], [41] and does not endogenously express inhibitory receptors for HLA-C. YTS cells transfected to express the inhibitory receptor KIR2DL1 (YTS-KIR2DL1) can be inhibited by expression on target cells of the KIR2DL1 ligand HLA-Cw6 [42]. To allow us to vary the size of HLA-Cw6 without confounding effects on peptide loading and HLA-Cw6 assembly we expressed it as a single-chain trimer fusion protein (hereafter termed Cw6) comprising a peptide, β_2_-microglobulin, and the HLA-Cw6 heavy chain joined by flexible glycine/serine linkers [43]. The size of Cw6 was varied, as with MICA, by inserting Ig domains from CD2, CD22, or CD4 into the stalk region linking the transmembrane and extracellular domains (Figure 3A). Transfectants were selected to express these constructs at comparable (high and low) levels on 721.221 cells (hereafter termed 221), EBV-transformed B cells which lack endogenous class I MHC protein expression (Figure 3A) [44].

Expression of normal-length Cw6 on 221 cells strongly inhibited killing by YTS/KIR2DL1 cells (Figure 3B). In contrast, elongated forms of Cw6 were less effective inhibitors, with the extent of inhibition progressively decreased with increasing Cw6 length (Figure 3B). Similarly, whereas normal-length Cw6 strongly inhibited IFN-γ secretion, elongated forms of Cw6 were far less inhibitory, even when expressed at high levels (Figure 3C). Similar results were obtained when intracellular IFN-γ was measured (Figure S3).

### Elongated Cw6 can bind to KIR2DL1

**Figure 4:**
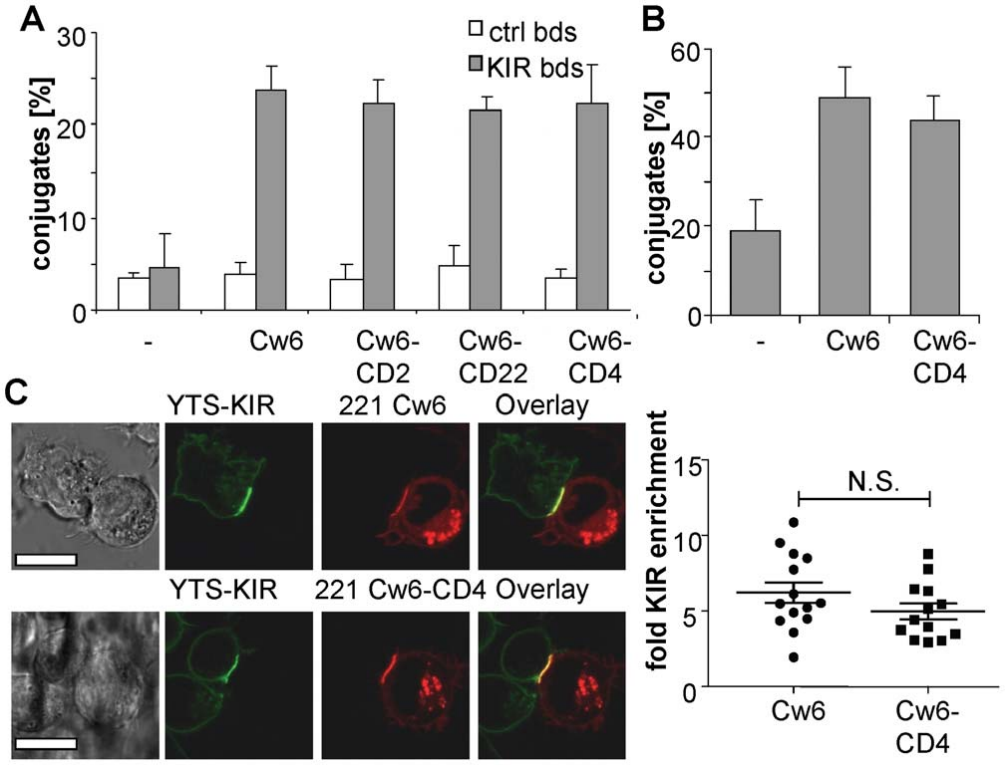
Elongated HLA-Cw6 binds KIR2DL1. (A) Binding of KIR2DL1 beads. 221 cells expressing the indicated form of Cw6 were incubated with fluorescent beads coated with KIR2DL1-CD4d34 or CD4d34. Bead binding was analysed by flow cytometry and expressed as percentage of cells forming conjugates to bead. A representative of two experiments is shown, the error bars represent the SD of triplicates. (B) P815 cells expressing normal-length or elongated Cw6 were allowed to form conjugates with equal numbers of KIR2DL1-expressing NKL cells for 30 min. The number of conjugates was determined as in Figure 2B. (1-tailed t-test: p = 0.025 for P815-Cw6 vs. P815, p = 0.037 for P815-Cw6-CD4 vs. P815 (C) Clustering of Cw6 and KIR2DL1 in YTS-221 immune synapses. 221 cells expressing the indicated Cw6-mCherry were allowed to form contacts with KIR2DL1-GFP -expressing YTS cells for 30 min and imaged live by confocal microscopy. Clustering was quantified as fold-enrichment of GFP brightness within the contact area versus outside the contact area. Each point represents an individual cell/cell contact with the mean and SD shown as lines, p = 0.11 (2-tailed t-test). The scale bar represents 10 µm.

One possible explanation for elongated Cw6 being less effective at inhibiting NK cell responses is that it could simply be unable to bind KIR2DL1. To test for this, we compared the binding of KIR2DL1-coated fluorescent beads to 221 cells expressing the various forms of Cw6. KIR2DL1-coated beads bound at comparable levels to cells expressing wild type and elongated Cw6 both in the absence (Figure 4A) and presence (Figure S4) of a titrated blocking antibody, indicating that elongation of Cw6 does not disrupt its interaction with KIR2DL1.

We next examined whether elongated Cw6 could bind KIR2DL1 in the context of an intercellular contact. To do this we examined conjugate formation between KIR2DL1-expressing NKL cells and P815 cells expressing normal-length or elongated Cw6. Expression of normal-length Cw6 on P815 cells resulted in a substantial increase in conjugate formation, with over 50% of NKL cells forming conjugates (Figure 4B). A similar level of conjugate formation was seen with Cw6-CD4, indicating effective binding of KIR2DL1 at the IS (Figure 4B).

As a final test of whether elongation of Cw6 affected KIR2DL1 binding we used live-cell confocal microscopy to visualize the accumulation of KIR2DL1-GFP and Cw6-mCherry at the interface between YTS and 221 transfectants (Figure 4C). This revealed that both normal-length and CD4-elongated Cw6 accumulated to similar extents and induced a similar level of accumulation of KIR2DL1; KIR2DL1 was enriched 6.2±2.0 fold (n = 14) at the IS with target cells expressing Cw6 and 5.0±1.5 fold (n = 13) for target cells expressing Cw6-CD4 (Figure 4C). The fact that KIR enrichment is not significantly different for elongated or normal-length Cw6 (t-test p>0.1) provides further evidence that both normal-length and elongated Cw6 are similarly able to bind KIR2DL1 at immune synapses.

### Segregation of Cw6 by size at the immune synapse

**Figure 5:**
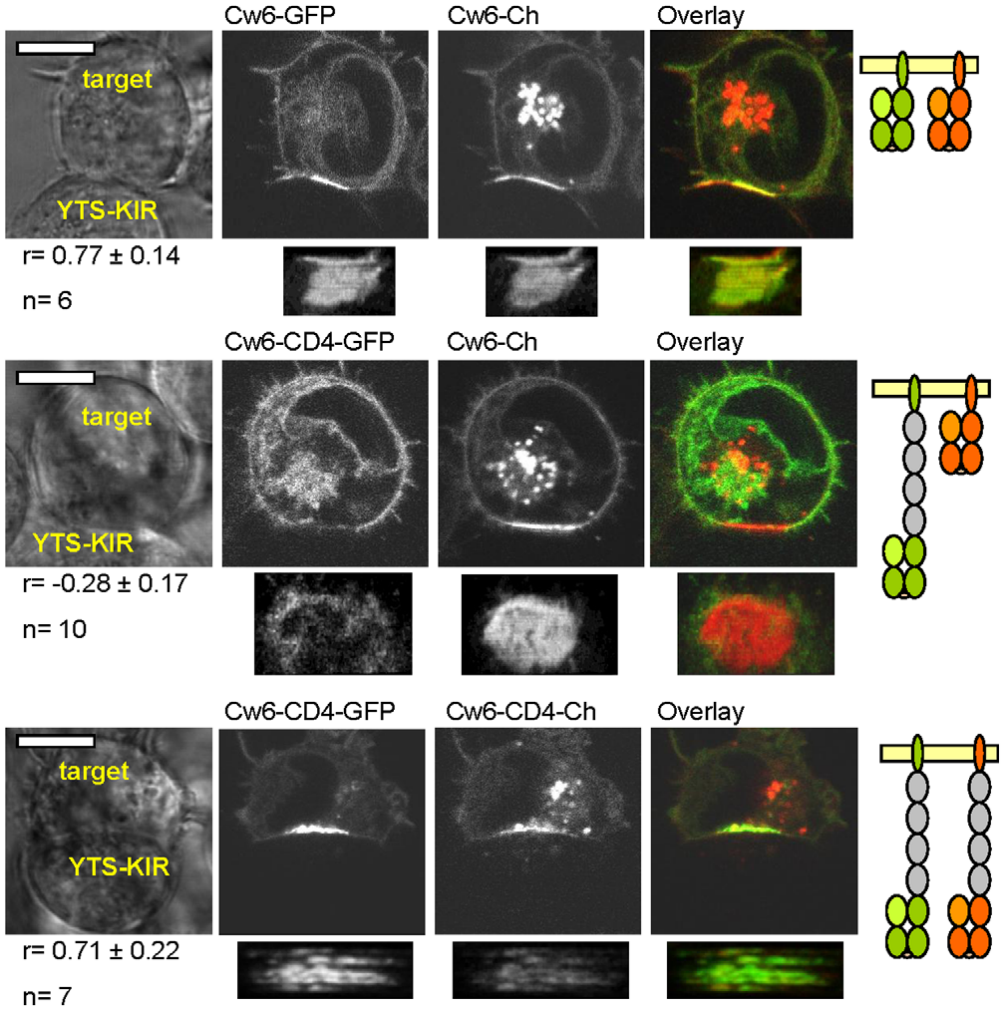
Size differences drive segregation of different length variants of HLA-Cw6 within immune synapses. 221 cells double-transfected with normal-length or CD4-elongated forms of Cw6 fused to either GFP (green) or mCherry (red) were incubated for 30 min with KIR2DL1-expressing YTS transfectants and conjugates were imaged by live-cell confocal microscopy. En-face views of the synapse were reconstructed and the Pearson correlation coefficient (r) for GFP and mCherry fluorescence were determined. Mean ± SD of the total number of conjugates is shown. The scale bar represents 10 µm.

We next tested whether Cw6 and Cw6-CD4 segregate or co-localise within the IS. We expressed normal-length and/or elongated Cw6 fused to different fluorescent proteins in 221 cells and visualized by confocal microscopy their location at inhibitory synapses with YTS-KIR2DL1 (Figure 5). Strikingly, elongated Cw6-CD4-GFP, segregated from normal-length Cw6-mCherry (Pearson coefficient r = −0.28±0.17 (SD), n = 10; Figure 5). Interestingly, the longer Cw6 tended to accumulate at the periphery of the IS (Figure S5). Importantly, normal-length Cw6-GFP and Cw6-mCherry co-localised at the IS, confirming that the type of the fluorescent tag itself did not affect segregation (Pearson coefficient r = 0.77±0.14, n = 6; Figure 5). Finally, elongated Cw6-CD4-GFP co-localised with Cw6-CD4-mCherry (r = 0.71±0.22, n = 7). These results demonstrate that differences in size are sufficient to drive segregation of proteins within the IS.

### Matching activating and inhibitory receptor/ligand size is important for signal integration

**Figure 6:**
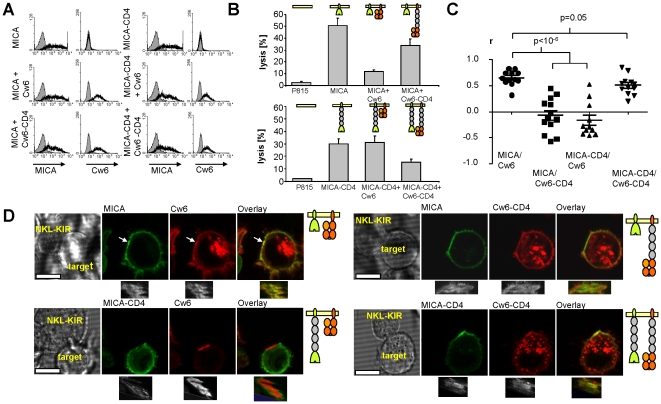
The size of Cw6 and MICA influences their localization within the synapse and the integration of their receptor signals. (A) A panel of P815 transfectants were produced expressing matching levels of normal-length or elongated MICA and Cw6. (B) NKL cells expressing the inhibitory receptor KIR2DL1 were mixed with P815 cells (E:T 20∶1) and cytotoxic activity determined as in Figure 1B. To augment cytotoxicity triggered by elongated MICA, IL-2 (100 U/ml) and IL-12 (10 ng/ml) were added exogenously. Data shown is representative of three independent experiments (mean ± SD of triplicates). (C, D) Organization of normal-length and elongated Cw6 and MICA in immune synapses. 221 cells expressing normal-length or elongated Cw6-mCherry (red) and MICA-GFP (green) were incubated with KIR2DL1-expressing NKL cells for 30 min before imaging by live-cell confocal microscopy. Scale bar represents 10 µm. From acquired image stacks, en-face views of the contact regions were reconstructed and the Pearson correlation coefficients (r) between GFP and mCherry fluorescence were determined. Data points represent individual synapses and the mean and SD are shown (p = 4.1×10^−7^ for MICA plus Cw6 vs. MICA plus Cw6-CD4; 1.3×10^−7^ for MICA plus Cw6 vs. MICA-CD4 plus Cw6; 0.05 for MICA plus HLA vs. MICA-CD4 plus Cw6-CD4 (2-tailed t-test).

Our finding that size-compatibility may be required for colocalisation raised the possibility that the elongation of HLA-Cw6 abrogates its inhibitory effect on NK cell activation because it prevents colocalisation of engaged KIR2DL1 with the engaged activating receptor(s) within the IS. We tested this possibility by varying the dimensions of both activating and inhibitory ligands simultaneously. Specifically, we compared lysis by KIR2DL1-expressing NKL cells of P815 cells expressing different combinations of normal-length or elongated MICA and Cw6 at matching levels of expression (Figure 6A). As shown previously, expression of normal-length MICA on P815 cells provoked killing by NKL cells (Figure 6B). Simultaneous expression on these cells of normal-length Cw6 was able to inhibit killing more effectively than expression of elongated Cw6 (Figure 6B). We next examined the effect of varying Cw6 length when MICA is elongated. Since elongation of MICA abrogates NKL activation (see Figure 1C, E) we augmented NKL activation by adding IL-2 and IL-12 to these assays. Under these conditions elongated MICA elicited killing, albeit more weakly that normal length MICA (Figure 6B). In contrast to the results obtained with normal length MICA, in P815 cells expressing elongated MICA simultaneous expression on normal length Cw6 did not inhibit killing, whereas expression of elongated Cw6 was able to inhibit killing (Figure 6B). Thus optimal inhibition by Cw6 of MICA-mediated killing was observed when Cw6 and MICA where matched in size.

Similar results were obtained using IFN-γ secretion as a readout of NKL cell activation (Figure S6A). Simultaneous expression of normal-length Cw6, but not elongated Cw6, was able to inhibit IFN-γ secretion induced by normal-length MICA (Figure S6A). Indeed, elongated Cw6 actually augmented IFN-γ secretion induced by MICA (Figure S6A). Conversely, normal length Cw6 but not elongated Cw6 augmented IFN-γ secretion induced by elongated MICA (Figure S6A). These positive effects probably arises from the greatly improved adhesion of NKL cells to P815 cells mediated by the KIR2DL1 interaction with normal-length or elongated Cw6 (Figure S6B), and may be particularly evident here as NKL and P815 cells are from different species and so lack endogenous adhesion receptor/ligand pairs. Furthermore, the secretion of cytokines is more dependent on the stability of the intercellular contact than target cell killing [45].

Taken together, these results indicate that the ligands MICA and Cw6 need to be matched in size for appropriate integration of positive and negative signals from their respective receptors. A plausible explanation for this is that the inhibitory KIRL2DL1-Cw6 complex needs to span the same distance as the activating NKG2D-MICA complex in order to colocalise with it. To examine this directly we used confocal microscopy to determine the distribution of normal-length and elongated forms of MICA-GFP and Cw6-mCherry within IS between KIR2DL1-expressing NKL and 221 cells (Figure 6C). Normal-length HLA-Cw6 co-localised with normal-length MICA (Pearson's correlation coefficient, r = 0.65±0.14 (SD), n = 12) whilst elongated MICA-CD4 segregated from normal-length Cw6 (r = −0.16±0.33, n = 11). Conversely elongated Cw6-CD4 co-localised with elongated MICA-CD4 (r = 0.51±0.19, n = 12) but not with normal-length MICA (r = −0.07±0.33, n = 13). This indicates that co-localization of activating and inhibitory receptors within the IS is dependent on matching ligand/receptor complex dimensions.

## Discussion

The ectodomains of leukocyte cell surface molecules vary greatly in size [21], [46]. Here, we set out to test for the first time whether the size of ligands for activating and inhibitory NK cell receptors influences NK cell responses. We first tested the functional consequence of elongating the extracellular domain of MICA by insertion of 2–4 domain spacers. While normal-length MICA was sufficient to trigger activation of NKL or primary NK cells, elongated forms of MICA were less effective, and the functional response was inversely correlated with MICA length. We showed that elongation did not abrogate NKG2D binding to MICA but did abrogate early signaling, as measured by SLP-76 phosphorylation. Taken together these findings suggest that elongation of MICA abrogates NKG2D triggering. The kinetic-segregation model predicts that elongation of a receptor/ligand complex would abrogate signaling because the increased intermembrane distance leads to less effective segregation of the engaged receptor from tyrosine phosphatases with large ectodomains. This is consistent with very recent data showing that elongation of mouse ligands for NKG2D also abrogated their efficacy in triggering NK cell activation [47]. Thus, the results presented here, showing that elongation of MICA abrogates NKG2D triggering, is consistent with NKG2D signaling involving the kinetic-segregation mechanism.

There are many activating receptors other than NKG2D expressed on NK cells, with 16 identified in humans [2]. While they vary in structure they have in common the fact that they are small and, where identified, their cell-surface ligands are also small [2]. Also, with the probable exception of DNAM-1 (CD226), they all signal by tyrosine phosphorylation of their cytoplasmic domains or the cytoplasmic domains of associated signaling subunits such as DAP10, DAP12 or CD3ζ, and this phosphorylation is thought to be mediated Src-like tyrosine kinases [2]. Thus, taken together with our finding that activation through NKG2D is inhibited by elongation its ligand, kinetic-segregation might be important for signaling by many if not all activating NK receptors.

We next investigated whether recognition by an inhibitory NK cell receptor was also dependent on ligand size. To do this we expressed a single-chain trimer form of HLA-Cw6 (Cw6) incorporating a peptide predicted to be compatible with KIR2DL1 binding. We used the single chain trimer to rule out any effect of elongation on peptide loading, as this could indirectly affect KIR recognition [48], [49]. Expression of normal-length Cw6 on 721.221 cells inhibited killing by KIR2DL1-expressing YTS cells. Elongation of Cw6, while not affecting binding to KIR2DL1, reduced its ability to inhibit NK cell-mediated lysis, with inhibition broadly inversely correlated with Cw6 length. It has also recently been reported that elongation of a mouse MHC class I molecule on target cells abrogates its inhibitory effect on mouse NK cell killing [47]. The inhibitory effect of KIR2DL1 requires phosphorylation of cytoplasmic tyrosine residues. One possible mechanism by which elongation of Cw6 abrogates KIR2DL1 function is that it inhibits signaling. Given the small size of the KIR2DL1-Cw6 complex, and the fact that signaling through KIR2DL1 is initiated by tyrosine phosphorylation of its cytoplasmic domain ITIM motif by Src kinases, it is plausible that KIR2DL1 signals through the kinetic-segregation mechanism. An alternative possibility is that KIR2DL1 is phosphorylated by Src kinases associated with other nearby small receptors, and that elongation of Cw6 inhibits signalling by preventing colocalization of KIR2DL1 with these receptors. Further studies are required to distinguish between these mechanisms.

Our finding that normal-length and elongated Cw6 spontaneously segregated at the IS shows that size differences alone are sufficient to drive segregation of receptor/ligand complexes in the IS. It has previously been shown that HLA-Cw6 segregated from regions enriched in ICAM-1 in a manner largely independent of cytoskeletal or ATP-dependent processes [17], [37], [50]. In addition, proteins were seen to segregate at synapses when expressed in insect cells thought not to express other ligands for human NK cells [51]. Our finding that differences in the molecular size alone are sufficient to drive segregation at the NK IS provides an explanation for these observations.

Inhibitory NK receptors such as KIR2DL1 abrogate NK cell activation by recruiting to the plasma membrane tyrosine phosphatases such as SHP-1 and SHP-2 and the inositol lipid phosphatases SHIP, where they directly oppose or reverse phosphorylation triggered by engagement of activating receptors [2]. This suggests that for inhibitory receptors to be effective they would need to co-localise with engaged activating receptors in the IS. This is supported by a recent study of NK activation on surfaces presenting micropatterned NK receptor ligands [52]. Our observation that differences in size alone result in segregation suggests that inhibitory receptor-ligand complexes would need to be similar in size to activating receptor-ligand complexes to colocalise with them and inhibit the activating signals. To test this hypothesis, we used cells expressing both normal-length or elongated Cw6 *and* normal-length or elongated MICA, in all 4 possible combinations. Our results confirmed that matched sizes of MICA and Cw6 proteins were important for co-localization within the IS and for signal integration; Cw6 and MICA colocalised best when they were similar in size. Similarly, normal sized Cw6 could only inhibit activation by normal-length MICA, while elongated Cw6 could only inhibit stimulation by elongated MICA. Thus, optimal signal integration between NKG2D and KIR2DL1 requires that NKG2D/MICA and KIR2DL1/Cw6 complexes are matched in size, probably because this is required for their colocalisation within the IS.

Our demonstration of the functional importance of matching dimensions of NKG2D/MICA and KIR2DL1/Cw6 raises the question as to whether this is required for signal integration between other activating and inhibitory NK receptors. In support of this the available structural information suggests that inhibitory receptor/ligand complexes all span similar dimensions (10–15 nm), despite significant variation in inhibitory receptor structure and length. For example although the LIR-1 (ILT-2) receptor has 4 Ig domains compared with the 2 Ig domains of most KIR molecules, it binds the membrane-proximal rather than the membrane-distal portion of MHC class I molecules [53], so that LIR-1/MHC class I and KIR2D/MHC class I complexes span similar distances. Furthermore, whereas KIR3D receptors have one more Ig domain than KIR2D receptors the two membrane-proximal Ig domains of both receptors are involved in binding [54], and so KIR3D-MHC class I and KIR2D-MHC class I complexes span a similar size. The similarity in the dimensions of activating and inhibitory NK receptor-ligand complexes, despite variation in primary structure and mode of binding, strongly suggests that this is of functional importance.

In conclusion, we have shown that elongation of the NKG2D ligand MICA abrogates NKG2D signaling, consistent with a role for the kinetic-segregation mechanism in signal transduction through NK receptors. We have also shown that the dimensions of complexes KIR2DL1-HLA-Cw6 and NKG2D-MICA need to be matched for optimal signal integration and colocalization in the NK cell IS. Our results suggest that size-dependent organisation of molecules within immune synapses is an important general principle for immune cell recognition.

## Materials and Methods

### Cell lines

The HLA-A/B/C negative human B-cell line 721.221 [55] [subsequently abbreviated as 221], the murine mastocytoma line P815 (ATCC, catalogue number TIB-64), and the EBV-immortalized human NK cell line YTS [42] were cultured in DMEM supplemented with 10% heat-inactivated FCS, 100 µg/ml streptomycin, 100 U/ml penicillin (all Invitrogen, Paisley, UK). The human NK cell leukemia line NKL [56], was cultured in RPMI-1640 supplemented with 10% heat-inactivated FCS, 100 µg/ml streptomycin, 100 U/ml penicillin, L-glutamine, β-mercaptoethanol, non-essential amino acids (all Invitrogen) and 100 U/ml IL-2 (Roche, Welwyn, UK). Primary human NK cells were isolated from healthy donor blood by negative magnetic selection (Miltenyi, Bergisch Gladbach, Germany) and cultured as described previously [57]. Primary NK cells were stimulated in 200 U/ml IL-2 for 4–5 days before the functional assays unless stated otherwise. Ethical approval was obtained from the Riverside Health Centre Ethics Committee (London, UK) and written informed consent was obtained from donors before obtaining and using their cells.

### Fusion protein constructs

Constructs encoding MICA and a single chain trimer version of HLA-Cw6 (hereafter termed Cw6) were generated in pcDNA3.1^+^ using a similar approach to that previously described [30], [31]. The junctions between MICA or Cw6 and C-terminal GFP/mCherry were (single letter amino acid code) EGA**ADPPVAT**MVSKG and ACKA**GGRS**MVSKG, respectively (junctional sequences underlined). The sequences preceding the additional junctional sequences are the C-termini of MICA and Cw6, and the sequence following them are the identical N-termini of GFP or mCherry. To facilitate the insertion of protein spacers, in-frame BamHI sites were introduced immediately after Thr292 and Trp288 in the stalk regions of MICA and Cw6, respectively. This required the mutation to Asp of His293 and Glu289 in MICA and Cw6, respectively. Fragments encoding the ectodomains of human CD2 and CD4, and the three N-terminal Ig domains of the mouse CD22 ectodomain were generated and inserted into these BamHI sites, as previously described [30], [31].

To facilitate the transfer of these constructs into the lentiviral vector pHR-SIN-BX-IRES-Em, an MluI site was introduced 6 base pairs upstream of the Not I site. The fragments encoding normal-length and elongated MICA and Cw6 with C-terminal tags of GFP or mCherry were amplified using forward and reverse primers that added Xho I and Mlu I sites, respectively, and then inserted into the XhoI/MluI sites of pHR-SIN-BX-IRES-Em. NKL-KIR2DL1-CFP transfectants was generated as follows: First, EGFP in the pEGFPN1 vector was exchanged with mCFP as a BamHI/BsrGI fragment, resulting in pMCFPN1. Then, KIR2DL1 was subcloned into pMCFPN1 from pcDNA3.1-KIR2DL1-CGFP (a kind gift from M. Purbhoo) using the HindIII/BamHI restriction sites. KIR2DL1-CFP was then subcloned from pMCFPN1-KIR2DL1 into the pIB2 vector transferring a blasticidin resistance (a kind gift from M. Purbhoo).

### Expression of cell surface proteins

Normal-length and elongated versions of Cw6 and MICA fused to GFP or mCherry [58] were expressed in target cells using lentiviral transduction. For this, 70–80% confluent 293T cells in a 175 cm^2^ flask were transfected with 14 µg expression vector pHR-SIN-BX-IRES-Em; 21 µg Vpack 11 µg VSVG (Stratagene, La Jolla, CA, USA), in 10 µM polyethylenimine. Supernatants cultured from 293T transfectants were taken 2 days later and concentrated 20 times. 2×10^6^ 221 or P815 cells were transduced with 1 ml lentivirus-containing supernatant for 4 hours, then cultured in complete medium for another 2 days. NKL were transduced by retrovirus produced by transfection of the Phoenix packaging cell line and selected with blasticidin. Transduced cells were sorted by flow cytometry (FACS DiVa/Aria II, BD/DAKO) based on fusion protein fluorescence and/or surface antigen staining. Fusion proteins were detected using anti-MICA staining (R&D, Abingdon, UK), followed by anti-mouse Alexa633, or Cy5-labeled anti-HLA (clone W6/32, ATCC). Fc receptors of P815 cells were pre-blocked by adding 2 µg/ml anti-CD16/CD32 (Fc block, BD, Oxford, UK) prior to antibody staining.

### Functional and binding assays

For NK cell activation assays, sorted transfectants of matching expression levels of the different length MICA/HLA were selected. NK cell cytotoxicity was determined using standard [^35^S]-Met radioactive release assays for 5 h.

In order to validate the ability of the MICA-fusion proteins to engage NKG2D, transfected cells were analyzed using conjugation assays with NKG2D beads. For this, streptavidin beads (Bangs Laboratories, Fishers, IN, USA) were coated with biotinylated anti-human Fc (Sigma, 5 µg/ml in PBS +0.1% BSA) at 4°C for 1 h under agitation. After washing three times with PBS, beads were coated with 10 µg/ml NKG2D-Fc fusion protein, or 10 µg/ml MICA-Fc fusion protein as a negative control (both R&D), followed by labelling with additionally anti-mouse-Alexa 633 (Molecular Probes). Following three washing steps with PBS, the beads were mixed with the target cells in a 96-well plate to a final density of 10^6^ cells each, briefly spun down and allowed to form conjugates for 1 h before fixing with ice-cold paraformaldehyde (total concentration 1%), followed by flow cytometry.

A similar bead binding assay was used to check the ability of the normal-length and elongated Cw6-fusion proteins to bind KIR2DL1. KIR2DL1 was expressed as a fusion protein KIR2DL1-CD4d34 comprising the entire KIR2DL1 ectodomain followed by rat CD4 domains 3 and 4 and the BirA biotinylation tag added at the C-terminus [59]. The sequence at the junction between KIR2DL1 and the CD4 was GNPRHLHGSTSITAYK (KIR2DL1 underlined). KIR2DL1-CD4d34 and a control protein CD4d34 lacking the KIR2DL1 portion were expressed, biotinylated, and coated onto avidin fluorescent beads (Spherotech) as previously described [59]. KIR2DL1-CD4d34 or CD4d34-coated fluorescent beads (15 µL) were incubated with 0.5 * 10^6^ 221 cells expressing various Cw6 proteins at 4°C for 2 hours and bead binding to 221 cells was measured by flow cytometry. For antibody-mediated blocking of bead binding, antibodies against MICA (R&D, stock concentration 0.5 mg/ml), or KIR2DL1 (HP3E4 hybridoma supernatant) were added to the cell/bead mix diluted as indicated.

Cell conjugation assays were performed based on previous protocols [60]. For the analysis of NKL conjugation with P815 transfectants, NK cells were stained with the red fluorescent dye PKH26 (2 µM), target cells with the green fluorescent dye PKH67 (2 µM) (both Sigma-Aldrich, Dorset, UK). After washing, 50 µL of 2 * 10^6^ cells/ml each of NK cell and target cell were mixed in a 96 well V-bottom plate, briefly spun down, and incubated for the times indicated. Conjugation was stopped by adding ice-cold paraformaldehyde to a final concentration of 1%, and conjugates were analysed by flow cytometry. The percentage of PKH26-labeled NK cells forming conjugates with PKH67-labeled target cells was determined as the number of double-positive events [100*(NK cells in conjugate)/(total number of NK cells)] (FloJo software).

IFN-γ expression and secretion were quantified by intracellar cytokine staining and ELISA, respectively, using standard protocols (antibodies from BD bioscience). The assay for degranulation was performed as described previously [61]: 10^6^ target cells/ml were cocultured with a different ratio of effector cells in a 48-well plate. Control wells contained either NK cells alone or NK cells stimulated with PMA (2.5 µg/ml) and ionomycin (0.5 µg/ml). PE-conjugated anti-CD107a mAb (Santa Cruz Biotechnology) was added and incubated for 1 h at 37°C. The exocytosis inhibitor monensin was added to a final concentration of 6 µg/ml and the incubation continued for a further 4 hours. Cells were washed 2 times with PBS, and in some experiments NK cells were stained for CD2, before analysis by flow cytometry (FACSCalibur).

For the analysis of SLP-76 phosphorylation by Western Blotting, 10^6^ NKL cells were stimulated by 10^6^ control or MICA-transduced P815 cells for 2 minutes in a 37°C water bath. Pelleted cells were sonicated for 5 s, and the samples then analysed using SDS-PAGE gel and Western Blot. Membranes were stained with Ab against SLP-76 phospho-tyrosine 113 (Abcam, Cambridge, UK) (1∶1000), followed by peroxidase-conjugated secondary antibody (1∶4000), and visualized by X-ray film.

### Microscopy and Image Analysis

Protein localization at NK/target cell synapses was determined by imaging live cells at 37°C/5% CO_2_ in 8-well glass bottom chamberslides (Labtek) using an inverted laser-scanning confocal fluorescence microscope (SP5, Leica) equipped with a 63x 1.2 NA water immersion objective, and a resonance scanner. Laser wavelengths of 488 and 591 nm were used for the excitation of GFP and mCherry, respectively. En-face views of the intercellular contact were rendered from multiple optical slices (Volocity, Improvision, Waltham, MA, USA). Quantification of fluorescence at the intercellular contact, and the determination of Pearson correlation coefficient r for the GFP and mCherry channel at the IS were performed using ImageJ (National Institute of Health).

## Supporting Information

Figure S1 **MICA elongation affects NK cell activation.** (A) MICA elongation affects intracellular IFN-γ. NKL cells were incubated for 1 h with equal numbers of P815 cells expressing the indicated length variant (or normal-length) MICA before adding Brefeldin A (5 µg/ml) and incubating for a further 4 h. Cells were then fixed and permeabilized, stained with PE-conjugated IFN-γ mAb, and analysed by flow cytometry. The percentage of IFN-γ^+^ NK cells is shown. (B) MICA elongation affects degranulation. NKL cells were incubated for 5 h in the presence of monensin with equal numbers of P815 cells expressing the indicated MICA. Degranulation was measured by CD107a antibody staining. The percentage of CD107a-positive NK cells is shown. These data represent the mean and SD of two independent experiments.
(TIF)

Figure S2 **NKG2D-bead binding to MICA-expressing P815 cells in the presence of blocking antibody.** Fluorescent streptavidin beads (Spherotech) were functionalized with NKG2D as described for Figure 2A. The indicated P815 cells were pre-treated with a MICA blocking Ab (R&D) at the indicated dilution (stock solution 50 µg/ml) in PBS +5% mouse serum for 30 min. NKG2D-coated beads were then incubated with these cells for 1 h at room temperature the number of bead-conjugated cells determined by flow cytometry. The data are a representative of 2 independent experiments.
(TIF)

Figure S3 **Cw6 elongation abrogates inhibition of NK cell activation.** YTS-KIR2DL1 cells were co-incubated with 221 cells expressing the indicated variant of Cw6 and intracellular IFN-γ was measured as described in Figure S1A. Data represent the mean and range of two independent experiments.
(TIF)

Figure S4 **KIR2DL1-bead binding to Cw6-expressing 221 cells in the presence of blocking antibody.** Fluorescent streptavidin beads were functionalized with KIR2DL1-Fc (a kind gift from Peter Parham, Stanford), as described for Figure 2A, and then pre-incubated with the indicated dilution of HP3E4 (stock solution 1∶10 hybridoma supernatant). These KIR2DL1-coated beads were then incubated in PBS +5% mouse serum for 1 h at room temperature with the indicated 221 cells, and the number of bead-conjugated cells determined by flow cytometry. The data is representative of 2 independent experiments.
(TIF)

Figure S5 **Elongation of Cw6 increases clustering at the synapse periphery.** 221 cells expressing GFP-tagged versions of HLA-Cw6 heavy chain (i.e. wild type Cw6) or normal-length (Cw6) or elongated (Cw6-CD4) single chain trimer versions of HLA-Cw6 were incubated with YTS-KIR2DL1 cells for 30 min and conjugates imaged by confocal microscopy. The relative location of the GFP clusters was determined by dividing the distance between the cluster centre and synapse centre by the diameter of the synapse, plotted at the y-axis. Each point represents an individual cell/cell contact with the mean and SD shown as lines, * denotes p<10-5.
(TIF)

Figure S6 **The size of Cw6 and MICA influences the integration of their receptor signals.** (A) NKL cells expressing the inhibitory receptor KIR2DL1 were incubated with the indicated P815 cells for 16 h before supernatants were harvested and IFN-γ measured. To augment IFN-γ secretion triggered by elongated MICA, IL-2 (100 U/ml) and IL-12 (10 ng/ml) were added exogenously. Data shown is representative of three independent experiments (mean ± SD of triplicates). (B) P815 cells expressing the indicated molecules were incubated with equal numbers of KIR2DL1-expressing NKL cells for 30 min. The number of conjugates was determined as in Figure 2B.
(TIF)
