# Supplementary figures and images for: Matched Sizes of Activating and Inhibitory Receptor/Ligand Pairs Are Required for Optimal Signal Integration by Human Natural Killer Cells
Source: PLoS One. 2010 Nov 5;5(11):e15374. doi: 10.1371/journal.pone.0015374 (PMC3001952; doi:10.1371/journal.pone.0015374)

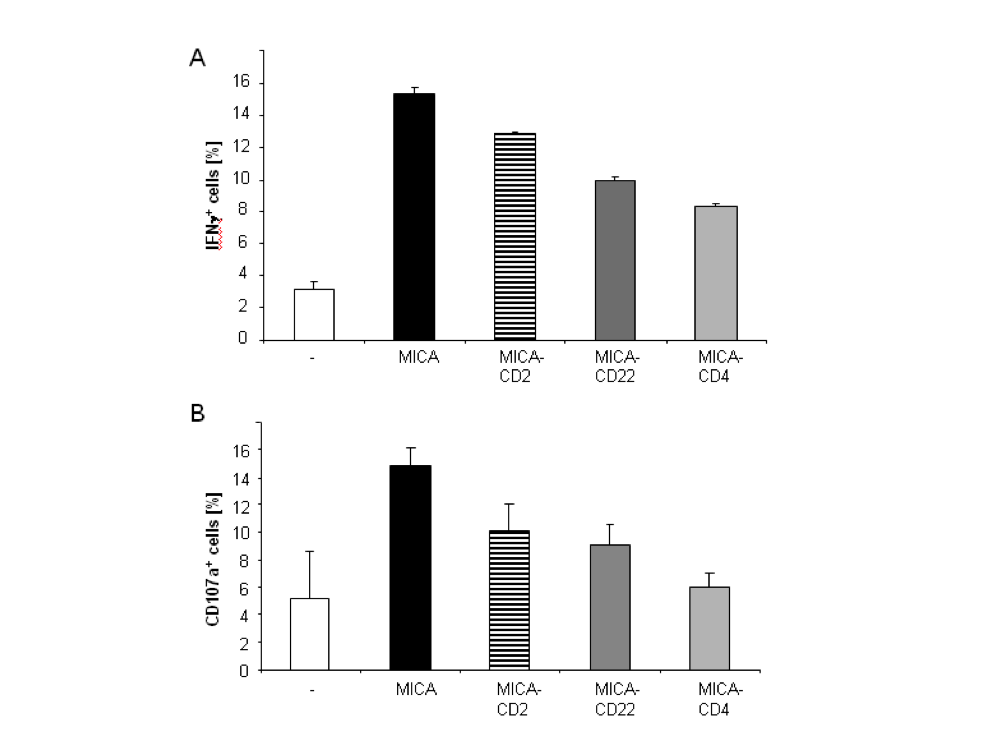

Supplement: Figure S1 — MICA elongation affects NK cell activation. (A) MICA elongation affects intracellular IFN-γ. NKL cells were incubated for 1 h with equal numbers of P815 cells expressing the indicated length variant (or normal-length) MICA before adding Brefeldin A (5 µg/ml) and incubating for a further 4 h. Cells were then fixed and permeabilized, stained with PE-conjugated IFN-γ mAb, and analysed by flow cytometry. The percentage of IFN-γ+ NK cells is shown. (B) MICA elongation affects degranulation. NKL cells were incubated for 5 h in the presence of monensin with equal numbers of P815 cells expressing the indicated MICA. Degranulation was measured by CD107a antibody staining. The percentage of CD107a-positive NK cells is shown. These data represent the mean and SD of two independent experiments. (TIF) [file pone.0015374.s001.tif]

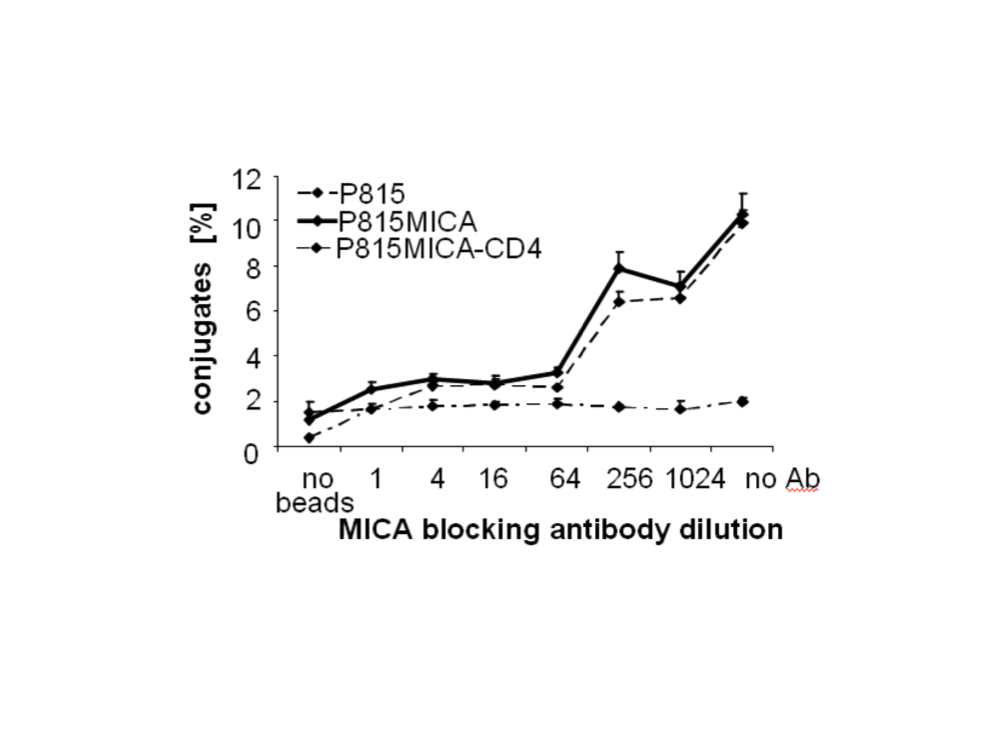

Supplement: Figure S2 — NKG2D-bead binding to MICA-expressing P815 cells in the presence of blocking antibody. Fluorescent streptavidin beads (Spherotech) were functionalized with NKG2D as described for Figure 2A. The indicated P815 cells were pre-treated with a MICA blocking Ab (R&D) at the indicated dilution (stock solution 50 µg/ml) in PBS +5% mouse serum for 30 min. NKG2D-coated beads were then incubated with these cells for 1 h at room temperature the number of bead-conjugated cells determined by flow cytometry. The data are a representative of 2 independent experiments. (TIF) [file pone.0015374.s002.tif]

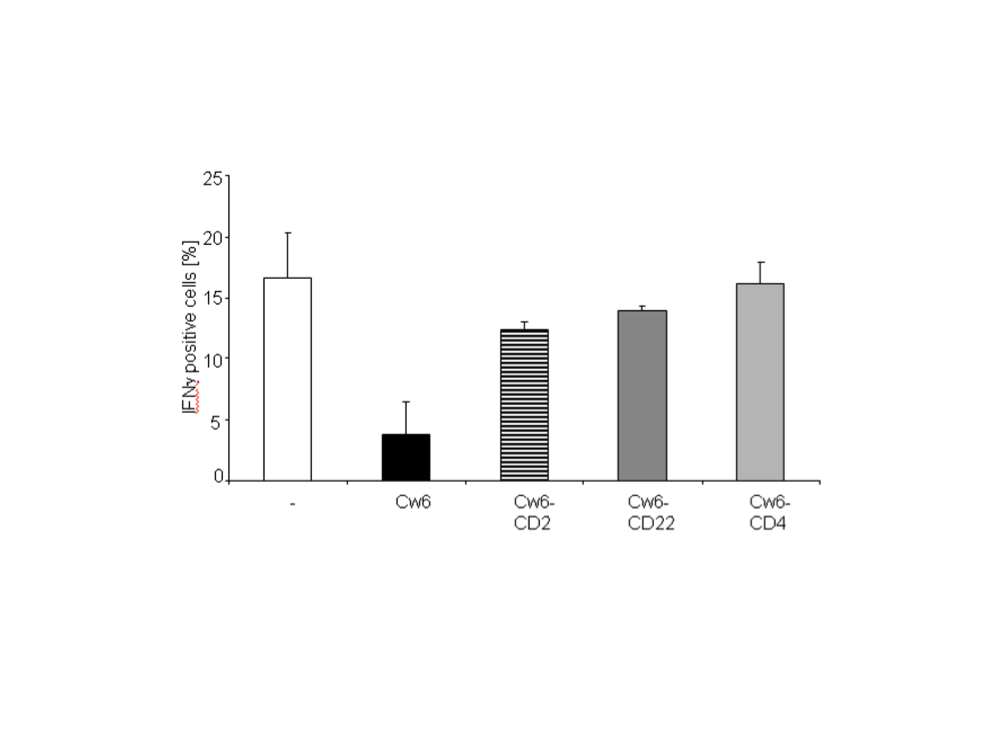

Supplement: Figure S3 — Cw6 elongation abrogates inhibition of NK cell activation. YTS-KIR2DL1 cells were co-incubated with 221 cells expressing the indicated variant of Cw6 and intracellular IFN-γ was measured as described in Figure S1A. Data represent the mean and range of two independent experiments. (TIF) [file pone.0015374.s003.tif]

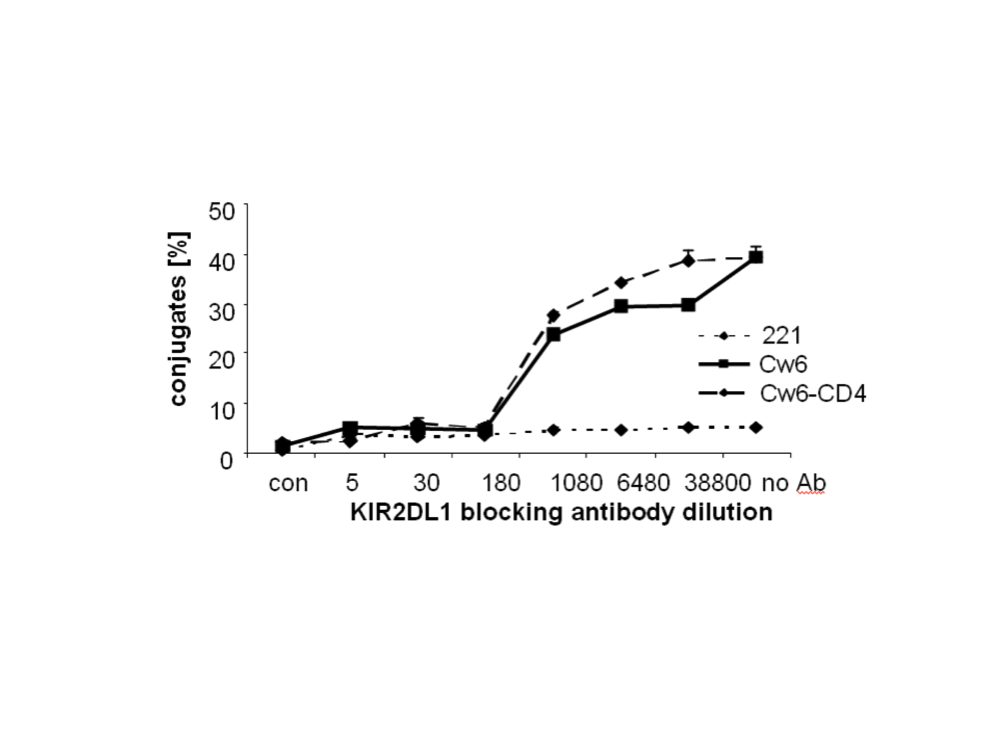

Supplement: Figure S4 — KIR2DL1-bead binding to Cw6-expressing 221 cells in the presence of blocking antibody. Fluorescent streptavidin beads were functionalized with KIR2DL1-Fc (a kind gift from Peter Parham, Stanford), as described for Figure 2A, and then pre-incubated with the indicated dilution of HP3E4 (stock solution 1∶10 hybridoma supernatant). These KIR2DL1-coated beads were then incubated in PBS +5% mouse serum for 1 h at room temperature with the indicated 221 cells, and the number of bead-conjugated cells determined by flow cytometry. The data is representative of 2 independent experiments. (TIF) [file pone.0015374.s004.tif]

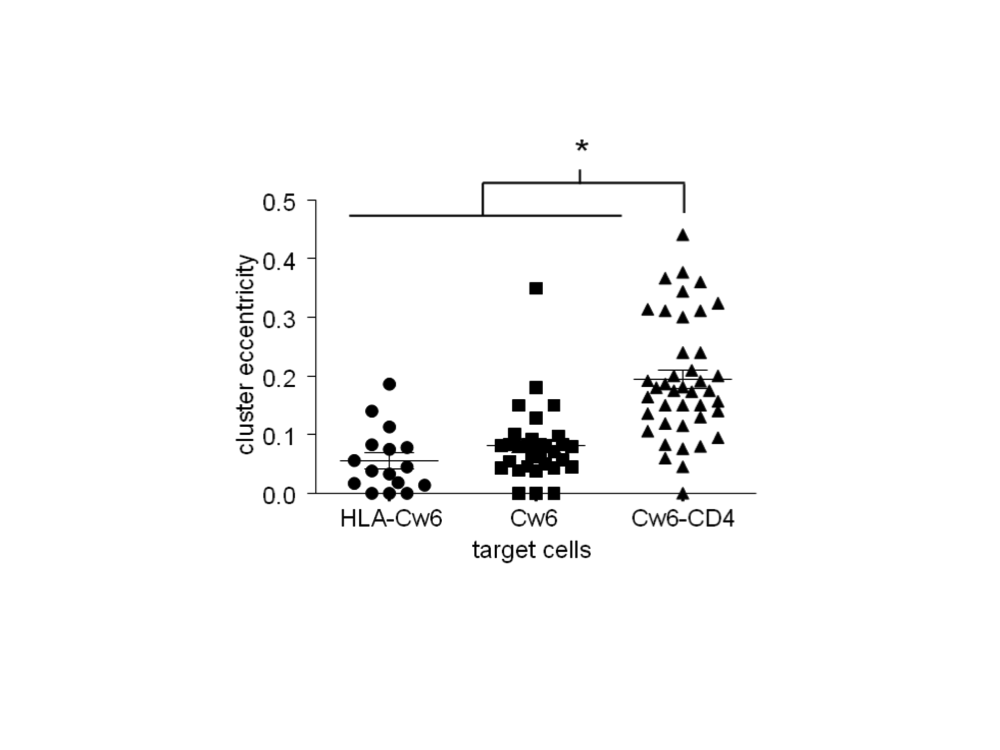

Supplement: Figure S5 — Elongation of Cw6 increases clustering at the synapse periphery. 221 cells expressing GFP-tagged versions of HLA-Cw6 heavy chain (i.e. wild type Cw6) or normal-length (Cw6) or elongated (Cw6-CD4) single chain trimer versions of HLA-Cw6 were incubated with YTS-KIR2DL1 cells for 30 min and conjugates imaged by confocal microscopy. The relative location of the GFP clusters was determined by dividing the distance between the cluster centre and synapse centre by the diameter of the synapse, plotted at the y-axis. Each point represents an individual cell/cell contact with the mean and SD shown as lines, * denotes p<10-5. (TIF) [file pone.0015374.s005.tif]

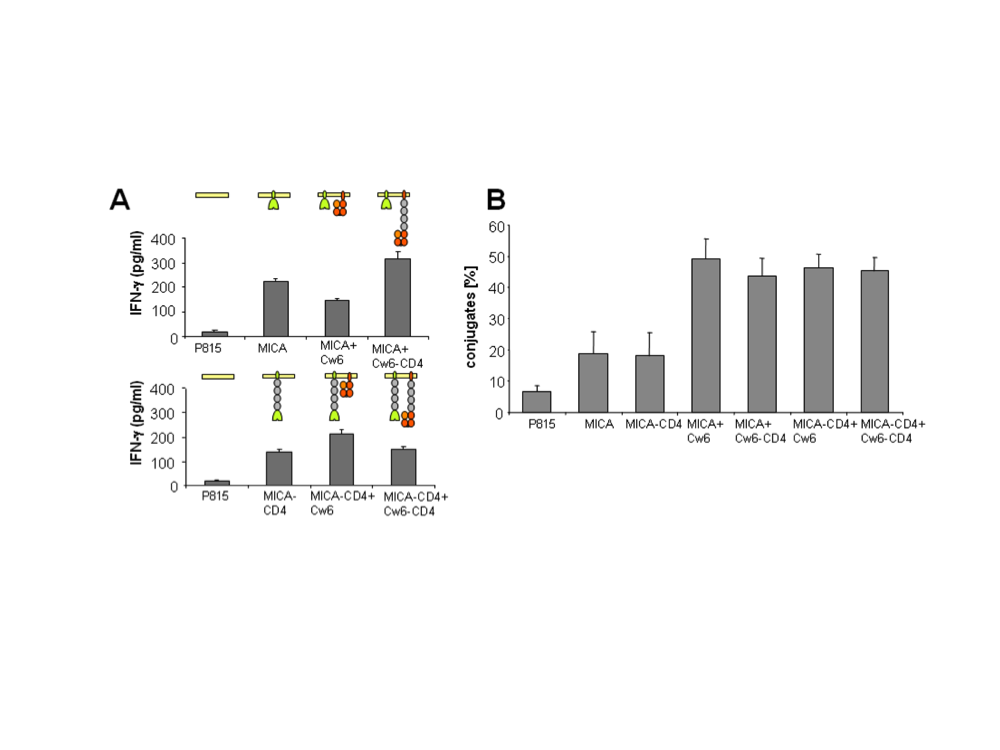

Supplement: Figure S6 — The size of Cw6 and MICA influences the integration of their receptor signals. (A) NKL cells expressing the inhibitory receptor KIR2DL1 were incubated with the indicated P815 cells for 16 h before supernatants were harvested and IFN-γ measured. To augment IFN-γ secretion triggered by elongated MICA, IL-2 (100 U/ml) and IL-12 (10 ng/ml) were added exogenously. Data shown is representative of three independent experiments (mean ± SD of triplicates). (B) P815 cells expressing the indicated molecules were incubated with equal numbers of KIR2DL1-expressing NKL cells for 30 min. The number of conjugates was determined as in Figure 2B. (TIF) [file pone.0015374.s006.tif]
